# Effect of Standardized Perioperative Management on EEG Indexes and Nerve and Limb Functions of Patients with Acute Cerebral Infarction Undergoing Mechanical Thrombectomy

**DOI:** 10.1155/2022/1686891

**Published:** 2022-09-26

**Authors:** Yu Gong, Jie Wang

**Affiliations:** ^1^Department of Interventional, Yantai Mountain Hospital, Yantai, 264003 Shandong, China; ^2^Department of Neurology, Shandong Provincial Hospital Affiliated to Shandong First Medical University, Jinan, 250021 Shandong, China

## Abstract

**Objective:**

To explore the application value of standardized perioperative management in mechanical thrombectomy for acute cerebral infarction.

**Methods:**

98 patients with acute cerebral infarction admitted to our hospital from January 2019 to January 2022 were selected as the study sample in this study, and all patients were given the standardized perioperative management. According to the interventional methods, they were divided into the thrombolytic treatment group (arteriovenous combined thrombolysis, *n* = 49) and mechanical thrombectomy group (mechanical thrombectomy, *n* = 49) to compare the nerve function, limb function, thrombolysis in myocardial infarction (TIMI) flow grade, symptomatic intracranial hemorrhage within 24 hours, acute vascular reocclusion, and the death status within 1 year and incidence of adverse events in 90 days of the two groups after treatment.

**Results:**

After treatment, the values of brain symmetry index (BSI) and power ratio indices (DTABR) in the two groups were obviously lower than those before treatment (*P* < 0.05), and the values of BSI and DTABR in the mechanical thrombectomy group were lower than those in the thrombolytic treatment group (*P* < 0.05). According to the statistical data of National Institutes of Health Stroke Scale (NIHSS) score in patients, the NIHSS scores of the two groups after treatment were visibly decreased (*P* < 0.05), while the NIHSS score in the mechanical thrombectomy group after treatment was lower than that in the thrombolytic treatment group (*P* < 0.05). The proportion of modified Rankin scale (mRS) score < 3 in the mechanical thrombectomy group was distinctly higher than that in the thrombolytic treatment group (*P* < 0.05). The proportion of TIMI flow grade ≥ 2 in the mechanical thrombectomy group was significantly higher than that in the thrombolytic treatment group (*P* < 0.05). The rate of symptomatic intracranial hemorrhage within 24 hours in the mechanical thrombectomy group was lower than that in the thrombolytic treatment group (*P* < 0.05), with the indistinctive difference between the two groups (*P* > 0.05). The incidence of acute vascular reocclusion in the mechanical thrombectomy group was markedly lower than that in the thrombolytic treatment group (*P* < 0.05). There was no significant difference in 1-year mortality between the two groups (*P* > 0.05). In the mechanical thrombectomy group, there were 1 case of gingiva bleeding, 1 case of hemorrhinia, and 2 cases of recurrent cerebral infarction in 90 days, with a total of 4 cases (8.16%), while in the thrombolytic treatment group, there were 4 cases of gingiva bleeding, 4 cases of hemorrhinia, and 15 cases of recurrent cerebral infarction in 90 days, with a total of 23 cases (46.94%), indicating that the incidence of adverse events in 90 days in the mechanical thrombectomy group was significantly lower than that in the thrombolytic treatment group (*P* < 0.05).

**Conclusion:**

The standardized perioperative management is effective in patients with acute cerebral infarction who were treated with arteriovenous combined thrombolysis or mechanical thrombectomy, which can improve the neurological function and physical function of patients. However, the mechanical thrombectomy has a better improvement effect on the neurological function and physical function of patients, with the relatively better safety, thrombolytic effect, and long-term prognosis.

## 1. Introduction

Acute cerebral infarction is a common acute disease in neurological department, and the symptoms of infarction occur due to the abnormal cerebral hemodynamics in patients. After onset of the disease, the reduction of regional blood volume in patients appears, thereby leading to the ischemic necrosis in local tissue, affecting the function of nerve cells and brain tissue, and making the pathological occurrence [1, 2]. The onset of acute cerebral infarction is urgent, which has a greater influence on the normal physiological function of patients, with the features of high mutilation rate and high mortality, and most patients have serious sequelae, which seriously affects the normal life. The epidemiological data show that the mortality in acute stage of cerebral infarction is about 5%-15%, and the disability rate in surviving patients is about 50%. For this disease, the main purpose of clinical treatment is to dredge the occluded blood vessel as soon as possible, thereby promoting the functional recovery of regional blood vessels and nerve cells. The cerebral vascular interventional therapy (arterial thrombolysis, mechanical recanalization, etc.) and intravenous thrombolytic therapy are the main methods of cerebral revascularization. Relatively speaking, the intravenous thrombolysis has a shorter therapeutic time window, but many patients still cannot get the thrombolytic therapy within the time window with the developed medical technology increasingly in today [3, 4]. With the continuous renovation of medical technology and equipment in China, cerebral vascular interventional therapy has developed rapidly. Arteriovenous combined thrombolysis not only reduces the dosage of thrombolytic agents but also extends the therapeutic time window. Related studies have shown that arteriovenous combined thrombolysis is also conducive to reducing the adverse reactions and is considered to be an effective method for rapid recanalization of occluded vessels. Mechanical thrombectomy is a method of reperfusion for acute cerebral infarction, which has the advantages of rapid recanalization in blood vessels and high vascular recanalization rate. However, there are few related clinical studies, and its safety and effectiveness still need to be explored constantly [5, 6]. In addition, complications such as intracranial hemorrhage and vascular injury caused by failure of canalization may occur in the process of interventional therapy, so that the combination of scientific and comprehensive standardized perioperative management can further prevent the occurrence of adverse events. Therefore, this study will deeply explore the application value of standardized perioperative management in mechanical thrombectomy for acute cerebral infarction.

## 2. Materials and Methods

### 2.1. Inclusion Criteria in Patients

(1) The onset time of acute cerebral infarction did not exceed 6 h. (2) The imaging manifestations did not show the large intracranial infarction in the early stage by CT examination. (3) The muscle strength of paralytic limbs was 0-3 grade. (4) Patients had the mild consciousness disorders. (5) The blood pressure was 180/100 mmHg. (6) The manifestations of neurological impairment in patients were less than 1 hour. (7) The family members were informed of this study and agreed to consult the patients' medical records.

### 2.2. Exclusion Criteria in Patients

(1) Patients with the severe liver and kidney dysfunction and cardiac insufficiency; (2) patients with the history of intracranial hemorrhage; (3) patients with active hemorrhage or severe trauma; (4) patients without the therapeutic indications of thrombolysis or mechanical thrombectomy; (5) patients who received the heparin anticoagulant therapy within 48 hours; (6) patients with the low coordination degree or missing visit subsequently; (7) patients with epilepsy at onset of disease; and (8) patients with the myocardial infarction in the near future.

### 2.3. Screening and Grouping of Patients

By means of the retrospective study, 98 patients with acute cerebral infarction admitted to our hospital from January 2019 to January 2022 were selected as the study sample in this study, and all patients received the standardized perioperative management. According to the interventional methods, they were divided into the thrombolytic treatment group (arteriovenous combined thrombolysis, *n* = 49) and the mechanical thrombectomy group (mechanical thrombectomy, *n* = 49). The study protocol was in line with the ethical and moral principles and approved by the hospital ethics committee.

### 2.4. Methods

#### 2.4.1. Standardized Perioperative Management

(1) Improvement of emergency operation processes to realize the efficient operation grading management. The notification of emergent surgery in paper version submitted on scene was canceled, and it was submitted online by the electronic medical records system. At the same time, the careful and feasible surgical plans were formulated to ensure the effective reception of electronic surgical application. The relevant information of emergency patients were recorded in detail, including the time of entering the operating room, the consultation hours of staff in operating room, the time of notifying the doctors, the arrival time of doctors, the operative surgery name, and the conditions of acceptance and treatment. The surgical green channel used in rescuing the emergency patients was established to realize the planned treatment of emergency patients in stages [7]. The efficiency of surgery grading management was improved by the information means, and the surgical procedures were recorded and supervised. (2) Real-time intraoperative monitoring. The real-time images of surgery were observed via the central monitor system of operating room. The surgical managers were able to monitor the intraoperative situations and the related data in real-time and immediately dispatch for the emergencies to ensure the perioperative safety. The segments such as entering and leaving the operating room, anesthesia and surgery, and entering and leaving the resuscitation room were displayed timely by controlling the important links and nodes in the perioperative period to provide information and data for ensuring the normal movement of surgery. At the same time, the application of anesthesia resuscitation room, induction room, and preoperative waiting room should be clarified, so as to ensure the sufficient materials and reasonable storage of goods [8]

#### 2.4.2. Arteriovenous Combined Thrombolysis

0.9% of sodium chloride injection at a dose of 100 ml added to urokinase (100 × 10^4^ *U*) was used completely by intravenous drip within 30 min. The thrombolytic steps were as follows. 1% of lidocaine at a dose of 10 ml was used for the local anesthesia of patients, and the right femoral artery was punctured using the modified Sedingger technique, with an inserted 6 F arterial catheter. Heparin (3000 U) was used by a drip chamber putted into the 6 F guiding catheter under the guidance of the guide wire, and the head end was sent to the pathological artery, vertebral artery, or carotid artery. The site of artery stenosis, stenosis degree, and case of collateral compensation were shown by photography [9]. The head of microwire or trensend microtubule was send to the distal thrombus according to the path graph, and the urokinase (10 × 10^4^ *U*) was given from the microtubule at 10 × 10^3^ *U*/min via pump. The head of catheter was inserted in thrombus by withdrawing, with the urokinase (10 × 10^4^ *U*) by injection. The catheter was withdrawn to the proximal thrombus, with the urokinase (10 × 10^4^ *U*) by injection, and the photography was performed 1time/10 min.

#### 2.4.3. Mechanical Thrombectomy

1% of lidocaine at a dose of 10 ml was used for the local anesthesia, and the right femoral artery of patients was punctured using the modified Sedingger technique, with an inserted 6 F arterial catheter. Heparin (3000 U) was used by a drip chamber putted into the 6 F guiding catheter under the guidance of the guide wire, and the head end was sent to the pathologic artery, vertebral artery, or carotid artery. The site of artery stenosis, stenosis degree, and case of collateral compensation were shown by photography. Under the roadmap, the Rebar18 microcatheter stent was sent to the delivery system under the guidance of Silver-speed-14 micro-wire or PT wire, and the head of the Rebar catheter was sent to the thrombus. Solitaire AB stent (4-20 mm) was sent from Rebar, with the retracement of Rebar and the release of stent. The stent was withdrawn after photography, and the thrombus that has been extracted was checked, while 30 m1 of blood in the guiding catheter was abstracted. The images were shown using photography after taking the thrombus in order to prevent the fallen thrombus into the cerebral artery again with the blood flow. The arterial catheter was removed, the vascular closure device in Abbott was used for hemostasis, and the puncture point was bandaged.

#### 2.4.4. Detection of Electroencephalogram

The routine electroencephalography (EEG) examination was performed using a digital video-EEG instrument, and the electrodes were placed according to the international 10-20 system to record the single-stage and dual-stage leads. The reference electrode was described when the patients had clear consciousness and full cooperation with the examination, with the detection time of 15-30 minutes. The patients' images and EEG were stored to select the ideal signal segment > 5 min for power spectrum analysis, including the brain symmetry index (BSI) and power ratio indices (DTABR). In addition, 5 ml of fasting venous blood was taken in the morning to centrifuge (1500 r/min and 15 min), and the serum was stored after centrifugation at -20°C for examination.

### 2.5. Observation Indices

Neurological function. The National Institutes of Health Stroke Scale (NIHSS) was used to evaluate the degree of neurologic impairment in patients, mainly from the 11 aspects of consciousness, eye movement, visual field, situation of facial paralysis, upper limbs motion, lower limbs motion, defective coordination, sensation, language, articulation disorder, and neglect syndrome. The different scores were corresponding to the severity of the disease, with a full score of 42. The higher the score, the more serious the symptoms of neurological deficits.

Physical function. The physical function of patients was evaluated using the modified Rankin scale (mRS) at 3 months after surgery. 0 point was no symptom. 1 point represented that patients had the symptoms but no obvious dysfunction, which could complete all daily work and life. 2 points indicated that patients with the mild disability were unable to complete all normal activities but could take care of their daily affairs without the help of others. 3 points indicated that patients with the moderate disability could walk independently, but they needed to be cared in part of life. 4 points represented that patients had the moderate to severe disability, which were unable to walk independently and needed to be cared in life. 5 points were severe disability, bedridden, urinary and fecal incontinence, and completely dependent on others in daily life.

TIMI flow grade. In the process of coronary angiography, the state of vascular lesions could be judged by the display of the contrast agent in the distal coronary arterial vessels, which was divided into 4 grades. Grade 0 referred to the distal blood vessels without the filling of contrast agent, suggesting that there was no blood perfusion in the distal end and indicating that the blood vessel might have complete occlusive diseases, thereby resulting that blood flow failed to pass completely. Grade 1 meant that the partial visualization of contrast agent was in the vascular stenosis site, but the contrast agent could not reach the distal blood vessel, indicating that the vascular stenosis was a severe lesion that was close to occlusion. Grade 2 represented that the contrast agent could fill the blood vessel with a visualization of blood vessel, but the developing speed was slower than the normal blood vessel, suggesting that there was a certain stenosis or lesion in the coronary artery. Grade 3 showed that the contrast agent could quickly and completely fill the blood vessel and displayed the vascular morphology, indicating that the blood flow was normal.

The symptomatic intracranial hemorrhage rate within 24 h and acute vascular reocclusion rate were counted. The waveform changes of *α*, *β*, *δ*, and *θ* in the two groups were observed by the EEG indicators, and the BSI and DTABR were compared between the two groups.

The incidence types of adverse events in patients between the two groups in 90 days were counted to calculate the incidence of adverse events.

### 2.6. Statistical Treatment

The software used for processing the data in this study was SPSS22.0, which mainly calculated the differences in data between the two groups, and GraphPad Prism 7 (GraphPad Software, San Diego, USA) was used for the chart production. The data included in the study were enumeration data and measurement data tested by *x*^2^ and *t* test, indicated by [*n* (%)] and ($\overset{¯}{x}\pm s$), which were in line with the normal distribution. The statistical results were *P* < 0.05, indicating the statistical differences between the two groups.

## 3. Results

### 3.1. General Information

There was no statistical difference in general information such as age, gender, sites of vascular occlusion, and onset time between the two groups (*P* > 0.05). See details in Table 1.

### 3.2. Detection of EEG

After treatment, the values of BSI and DTABR in the two groups were obviously lower than those before treatment (*P* < 0.05), and the values of BSI and DTABR in the mechanical thrombectomy group were lower than those in the thrombolytic treatment group (*P* < 0.05). See details in Table 2.

### 3.3. NIHSS Score and mRS Score

According to the statistical data of NIHSS score in Table 3, the NIHSS scores of the two groups after treatment were significantly lower (*P* < 0.05), while the NIHSS score in the mechanical thrombectomy group after treatment was lower than that in the thrombolytic treatment group (*P* < 0.05). According to the statistical results in Table 3, the proportion of mRS score < 3 in the mechanical thrombectomy group was significantly higher than that in the thrombolytic treatment group (*P* < 0.05).

### 3.4. TIMI Flow Grade

According to the statistical data in Table 4, the proportion of TIMI flow grade ≥ 2 in the mechanical thrombectomy group was significantly higher than that in the thrombolytic treatment group (*P* < 0.05).

### 3.5. Symptomatic Intracranial Hemorrhage within 24 Hours, and Acute Vascular Reocclusion and Death Status within 1 Year

**Figure 1:**
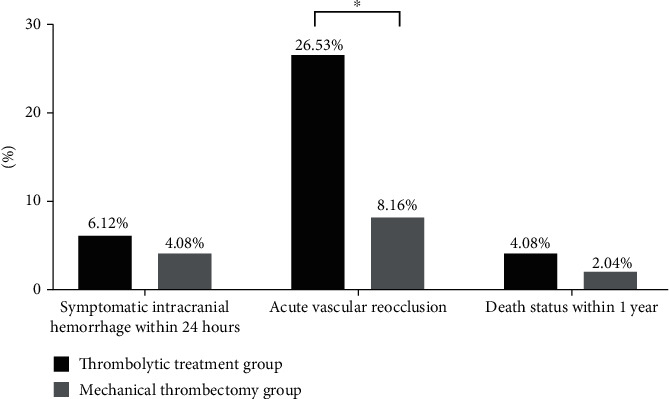
Symptomatic intracranial hemorrhage within 24 hours, acute vascular reocclusion and death status within 1 year. Notes. The horizontal coordinate represented the evaluation dimension, and the vertical coordinate represented the percentage (%). There were 3 cases with symptomatic intracranial hemorrhage within 24 hours, 13 cases with acute vascular reocclusion, and 2 dead cases within 1 year in the thrombolytic treatment group, while there were 2 cases with symptomatic intracranial hemorrhage within 24 hours, 4 cases with acute vascular reocclusion, and 1 dead case within 1 year in the mechanical thrombectomy group. ^∗^ represented a significant difference in the incidence of acute vascular reocclusion between the two groups (*X*^2^ = 5.765, *P* = 0.016).

The rate of symptomatic intracranial hemorrhage within 24 hours in the mechanical thrombectomy group was lower than that in the thrombolytic treatment group (*P* < 0.05), with the indistinctive difference between the two groups (*P* > 0.05). The incidence of acute vascular reocclusion in the mechanical thrombectomy group was markedly lower than that in the thrombolytic treatment group (*P* < 0.05). There was no significant difference in 1-year mortality between the two groups (*P* > 0.05). See details in Figure 1.

### 3.6. Incidence of Adverse Events in 90 Days

In the mechanical thrombectomy group, there were 1 case of gingiva bleeding, 1 case of hemorrhinia, and 2 cases of recurrent cerebral infarction in 90 days, with a total of 4 cases (8.16%), while in the thrombolytic treatment group, there were 4 cases of gingiva bleeding, 4 cases of hemorrhinia, and 15 cases of recurrent cerebral infarction in 90 days, with a total of 23 cases (46.94%), indicating that the incidence of adverse events in 90 days in the mechanical thrombectomy group was significantly lower than that in the thrombolytic treatment group (*P* < 0.05).

## 4. Discussion

Acute cerebral infarction is a common cerebrovascular disease with high mutilation rate and mortality, and the therapeutic key is early vascular recanalization. In recent years, with the development of clinical medical technology, the clinical treatment programs of patients with acute cerebral infarction have become more and more perfect, and mechanical thrombectomy is the most advanced interventional treatment for ischemic stroke in clinic, which has a more significant treatment effect especially for large artery occlusion. Clinical studies have shown that intravascular mechanical thrombectomy is an important method for rapid and efficient recanalization of acute aortic occlusive cerebral infarction, which can effectively improve the mRS scores of patients for 90 days [10, 11]. According to the Guidelines for the Diagnosis and Treatment of Acute Ischemic Stroke in China, the intravascular mechanical thrombectomy is recommended for patients with indications [12–14]. Similar to the mechanical thrombolysis, thrombolysis with thrombolytic agents can achieve the cerebral revascularization, reconstruct the cerebral blood flow, and save the ischemic brain tissue, showing that thrombolysis is the most effective and promising method for the treatment of acute cerebral infarction from this perspective. Among them, intravenous thrombolysis is easy to be accepted by patients because of the low requirements of technical equipment and low cost, but intravenous thrombolysis also has the risk of intracranial hemorrhage resulting in low vascular recanalization rate, high mortality, and high probability of early reocclusion after the thrombolysis [15, 16]. However, arterial thrombolysis has more advantages in some aspects compared with the intravenous thrombolysis, for example, the arterial thrombolysis can accurately determine the sites, degree of occlusion, compensation, and the cases of recanalization in occluded vessels by digital subtraction angiography, and the selectivity of local contact medication in thrombus is higher. Related studies have also pointed out that the vascular recanalization rate and safety of arterial thrombolysis are relatively high, but the intracranial hemorrhage transformation is the most serious complication of arterial thrombolysis, with a high mortality [17, 18]. The combination of the two thrombolytic methods has a better therapeutic effect than single method, which can integrate the advantages of the two methods and improve the efficiency and area of drug use, with a better thrombolytic effect. Compared with the arterial-venous thrombolytic treatment, mechanical thrombectomy with a late start can break and remove the thrombus, which greatly reduces the possibility of recurrence. It provides a new method and idea for clinical treatment of acute cerebral infarction and is also a new trend in the future treatment of acute cerebral infarction.

Surgical management is an administrative work with comprehensiveness and multidisciplinary coordination, especially for critically ill patients such as acute cerebral infarction. The scientific and modern perioperative management can be realized only via setting up the reasonable organizational structure and optimizing the management process constantly [19–21]. With the development of medical information technology, information means provide the possibility for the realization of meticulous surgical management. Our hospital actively explored the role of standardized perioperative management in strengthening the attention of surgical efficiency and management efficiency and ensuring the surgical effect and safety in order to strengthen the attention on perioperative management of patients with acute cerebral infarction [22–24]. Based on this, the following results were further obtained in this study. After treatment, the values of BSI and DTABR in the two groups were obviously lower than those before treatment (*P* < 0.05), and the values of BSI and DTABR in the mechanical thrombectomy group were lower than those in the thrombolytic treatment group (*P* < 0.05). BSI is a monitoring index of cerebral blood flow in common use during carotid endarterectomy, and its application value in diagnosis and prognosis monitoring of brain diseases has gradually become prominent in recent years. The analysis shows that BSI can reflect the difference in activity status of brain cells between the unilateral supply region and contralateral supply region when the patients have changes of cerebral hemodynamics, thereby reflecting the cases of infarction in patients and the change of cerebral blood flow during treatment. DTABR mainly reflects the change on the ratio of *δ* + *θ* wave and *α* + *β* wave. When the patients have infarction, the amplitude of the whole brain decreases, *α* wave disappears, *β* wave decreases, and the level of *α* + *β* wave decreases, while the level of *δ* + *θ* wave increases, resulting in the increase of DTABR value. Therefore, DTABR value has a higher application value for monitoring the curative effect of patients. The results showed that simple mechanical thrombectomy was more effective in improving the intracranial hemodynamics of patients with acute cerebral infarction and was beneficial to the recovery of brain function. According to the NIHSS scores in patients, the NIHSS scores of the two groups after treatment were visibly decreased (*P* < 0.05), while the NIHSS score in the mechanical thrombectomy group after treatment was lower than that in the thrombolytic treatment group (*P* < 0.05), which was consistent with the study of Hoorn et al. [25]. The proportion of mRS score < 3 in the mechanical thrombectomy group was distinctly higher than that in the thrombolytic treatment group (*P* < 0.05), further indicating that mechanical thrombectomy can promote the recovery of neurological function and physical function compared with the thrombolytic therapy. The proportion of TIMI flow grade ≥ 2 in the mechanical thrombectomy group was significantly higher than that in the thrombolytic treatment group (*P* < 0.05), and the rate of symptomatic intracranial hemorrhage within 24 hours in the mechanical thrombectomy group was lower than that in the thrombolytic treatment group, with the indistinctive difference between the two groups (*P* > 0.05), while the incidence of acute vascular reocclusion in the mechanical thrombectomy group was markedly lower than that in the thrombolytic treatment group (*P* < 0.05). The results suggested that mechanical thrombectomy had the best thrombolytic effect on patients with acute cerebral infarction. At the same time, the combination of standardized perioperative management was conducive to ensuring the perioperative safety of patients and was also an important guarantee for the smooth progress of mechanical thrombectomy. There was no significant difference in 1-year mortality between the two groups (*P* > 0.05). At present, the studies on mechanical thrombectomy are mostly in the active exploration period, and the observation results of long-term efficacy are different, which may be related to factors such as thrombectomy device and patient selection. The Solitaire AB stent used in this study is a self-expandable NiTi alloy material with the design of closed mesh, and one side is fully opened. Characterized by a good transport capacity and higher radial support force, the design combines the advantages of closed and open mesh, without the protrusion and deformation phenomenon, and the thrombus can be removed by pulling the stent after release. Combined with the standardized perioperative management, the short-term curative effect is better, and the long-term mortality is also lower than that in many previous studies.

In summary, the standardized perioperative management is effective in patients with acute cerebral infarction who were treated with arteriovenous combined thrombolysis or mechanical thrombectomy, which can improve the neurological function and physical function of patients. However, mechanical thrombectomy has a better improvement effect on neurological function and physical function of patients, with relatively good safety, thrombolysis effect, and long-term prognosis. It can be affirmed that standardized perioperative management combined with simple mechanical thrombectomy has a greater potential application value for patients with acute cerebral infarction, which can be prioritized in patients that meet the treatment indications. However, each method has certain limitations for the acute thrombolytic therapy of such patients. For the benefit of patients after opening the blood vessels, it is more dependent on the size of the penumbra and the core infarct region, so that the evaluation of early imaging data of patients should be emphasized.

## Figures and Tables

**Table 1:** Comparison of general information in patients between the two groups (*n* = 49).

Observation indices	Thrombolytic treatment group	Mechanical thrombectomy group	*X* ^2^/*t*	*P*
Age range	55-79	57-78		
Average age (years)	68.55 ± 4.82	68.37 ± 4.71	0.187	0.852
Gender			0.165	0.685
Male	26 (53.06)	28 (57.14)		
Female	23 (46.94)	21 (42.86)		
Sites of vascular occlusion				
M1/M2 segment of middle cerebral artery	12 (24.49)	13 (26.53)	0.054	0.817
Intracranial internal carotid artery	13 (26.53)	12 (24.49)	0.054	0.817
Involvement of middle artery and internal carotid artery	13 (26.53)	11 (22.45)	0.221	0.638
Vetebral-basilar artery	11 (22.45)	13 (26.53)	0.221	0.638
Average onset time (*h*)	4.05 ± 1.12	4.08 ± 1.01	0.139	0.890

**Table 2:** Statistics of EEG indexes between the two groups.

Groups	BSI value		DTABR	
	Before treatment	After treatment	Before treatment	After treatment
Thrombolytic treatment group	0.22 ± 0.04^∗^	0.14 ± 0.03	1.09 ± 0.28^∗^	0.82 ± 0.18
Mechanical thrombectomy group	0.23 ± 0.03^∗^	0.09 ± 0.02	1.10 ± 0.25^∗^	0.66 ± 0.15
*t*	1.400	9.707	0.186	4.780
*P*	1.165	<0.001	0.853	<0.001

Note. Brain symmetry index (BSI) and power ratio indices (DTABR). ^∗^ represented a significant difference in the same group before and after treatment (*P* < 0.05).

**Table 3:** Statistics of NIHSS score and mRS score in patients.

Groups	NIHSS score		mRS score	
	Before treatment	After treatment	<3 points	≥3 points
Thrombolytic treatment group	9.22 ± 3.20^∗^	7.14 ± 1.75	33 (67.35)	16 (32.65)
Mechanical thrombectomy group	9.27 ± 3.19^∗^	4.96 ± 1.11	46 (93.88)	3 (6.12)
*t*/*X*^2^	0.077	7.364	11.034	
*P*	0.938	<0.001	4.780	

Notes. The national institutes of health stroke scale (NIHSS) and modified Rankin scale (mRS). ^∗^ indicated an obvious difference in the same group before and after treatment (*P* < 0.05).

**Table 4:** Statistics of TIMI flow grade in patients.

Groups	Cases	≥2 grade	>3 grade
Thrombolytic treatment group	49	29 (59.18)	20 (40.82)
Mechanical thrombectomy group	49	47 (95.92)	2 (4.08)
*t*		18.990	
*P*		<0.001	

Notes. Thrombolysis in myocardial infarction (TIMI) flow grade.

## Data Availability

Data to support the findings of this study are available on reasonable request from the corresponding author.
